# Increase of ATP-sensitive potassium (K_ATP_) channels in the heart of type-1 diabetic rats

**DOI:** 10.1186/1475-2840-11-8

**Published:** 2012-01-18

**Authors:** Zhih-Cherng Chen, Yung-Ze Cheng, Li-Jen Chen, Kai-Chun Cheng, Yin- Xiao Li, Juei- Tang Cheng

**Affiliations:** 1 Department of Cardiology, Chi-Mei Medical Center, Yong Kang, Tainan City, 73101 Taiwan; 2 Department of Medical Research and, Chi-Mei Medical Center, Yong Kang, Tainan City, 73101 Taiwan; 3 Department of Emergency Medicine, Chi-Mei Medical Center, Yong Kang, Tainan City, 73101 Taiwan; 4 Institute of Basic Medical Sciences, College of Medicine, National Cheng Kung University, Tainan City, 70101 Taiwan; 5 Department of Psychosomatic Internal Medicine, Kagoshima University Graduate School of Medical and Dental Sciences, Kagoshima City 890-8520, Japan; 6 Department of Pharmacy, Chia Nan University of Pharmacy & Science, Jean-Tae, Tainan City, 71701 Taiwan

**Keywords:** Cardiac ATP-sensitive potassium channel, Gene expression, Insulin, Phlorizin, Diabetic rats

## Abstract

**Background:**

An impairment of cardiovascular function in streptozotocin (STZ)-diabetic rats has been mentioned within 5 days-to-3 months of induction. ATP-sensitive potassium (K_ATP_) channels are expressed on cardiac sarcolemmal membranes. It is highly responsive to metabolic fluctuations and can have effects on cardiac contractility. The present study attempted to clarify the changes of cardiac K_ATP _channels in diabetic disorders.

**Methods:**

Streptozotocin-induced diabetic rats and neonatal rat cardiomyocytes treated with a high concentration of glucose (a D-glucose concentration of 30 mM was used and cells were cultured for 24 hr) were used to examine the effect of hyperglycemia on cardiac function and the expression of K_ATP _channels. K_ATP _channels expression was found to be linked to cardiac tonic dysfunction, and we evaluated the expression levels of K_ATP _channels by Western blot and Northern blot analysis.

**Results:**

The result shows diazoxide produced a marked reduction of heart rate in control group. Furthermore, the methods of Northern blotting and Western blotting were employed to identify the gene expression of K_ATP _channel. Two subunits of cardiac K_ATP _channel (SUR2A and kir 6.2) were purchased as indicators and showed significantly decreased in both diabetic rats and high glucose treated rat cardiac myocytes. Correction of hyperglycemia by insulin or phlorizin restored the gene expression of cardiac K_ATP _in these diabetic rats.

**Conclusions:**

Both mRNA and protein expression of cardiac K_ATP _channels are decreased in diabetic rats induced by STZ for 8 weeks. This phenomenon leads to result in desensitization of some K_ATP _channel drugs.

## Background

Diabetes is a disease characterized by chronic hyperglycemia secondary to a reduction in the functional efficacy and/or a deficiency of insulin. In fact, patients with diabetes have a shorter life span and a lesser quality of life, mainly as a result of macrovascular and/or microvascular complications[1]. An impairment of cardiovascular function in streptozotocin (STZ)-diabetic rats has been mentioned within 5 days-to-3 months of induction [2].

ATP-sensitive potassium (K_ATP_) channels are expressed on cardiac sarcolemmal membranes, and can have effects on cardiac repolarization and contraction during physiological and pathophysiological conditions [3-5]. Sarcolemmal K_ATP _channels are composed of a pore-forming subunit (kir6.1 or kir6.2) and a sulfonylurea receptor (SUR1, SUR2A or SUR2B) [6]. Activation of K_ATP _channels plays an important role of cardio-protection during myocardial ischemia and hypoxia [7-9].

In the cardiac muscular cells, K_ATP _channel gating is highly responsive to metabolic fluctuations in the channel microenvironment[10]; the K_ATP _could act as sensor of cell energy metabolism. K_ATP _channel senses signals of cell energy metabolism in two ways. One is direct interactions between K_ATP _and cell metabolites, which will produce immediate and temporal effects on channel activities[11]; the other is regulation of K_ATP _genes expression by energy metabolism, this way can induce a delayed but much profound effect on channel quantity[12].

Cell energy metabolism regulates K_ATP _genes expression; alternations in the metabolism will lead to changes of the K_ATP _channel number[12]. High glucose leads to a marked decrease of *kir6.2 *mRNA level in isolated rat pancreatic islets as well as in the INS-1 beta cell line. This effect is reversed by exposure to low glucose[13]. Taken into together, investigation on the gene expression of cardiac K_ATP _might clarify the cardiac dysfunction during diabetes development.

In order to demonstrate the changes of cardiac K_ATP _channels in diabetic disorders, the present study employed the whole heart of diabetic rats induced by STZ injection for 8 weeks and neonatal rat cardiomyocytes. The alterations of cardiac K_ATP _channels in the protein and mRNA levels were employed as indicators.

## Methods

### Animals

Three-month-old male Wistar rats were housed in a temperature controlled room (25°C) with a 12-h dark and 12-h light cycle. Food and water were available at its pleasure. Diabetic rats were prepared by giving an intravenous (IV) injection of 60 mg/kg streptozotocin (STZ) (Sigma-Aldrich, Inc., Saint Louis, Missouri, USA), into the fasting rats. Animals were considered to be diabetic if they had plasma glucose concentrations of 20 mmol/l or greater in addition to polyuria and other diabetic features. All studies were carried out 2 weeks after the injection of STZ. The concentration of plasma glucose was measured by the glucose oxidase method using an analyzer (Quik-Lab, Ames, Miles Inc., Elkhart, Indiana, USA). The animal experiment was approved and conducted in accordance with local institutional guidelines for the care and use of laboratory animals in the Chi-Mei Medical Center (No. 100052307) and conformed with the Guide for the Care and Use of Laboratory Animals (Kilkenny C et al. Improving bioscience research reporting: the ARRIVE guidelines for reporting animal research. PLoS Biol 2010, Jun 29;8(6):e1000412), as well as the guidelines of the Animal Welfare Act.

### Cell cultures

Primary cultures of neonatal rat cardiomyocytes were prepared by modification of a previously described method [14]. Briefly, under anaesthesia with pentobarbital (30 mg/Kg), the heart tissue from a 1- to 2-day-old Wistar rat was excised then cut into 1-2 mm pieces and predigested with trypsin to remove red blood cells. The heart tissue was then digested with 0.25% trypsin and 0.05% collagenase. The dissociated cells were placed in uncoated 100 mm dishes and incubated at 37°C in a 5% CO2 incubator for at least 1 h to remove the non-myocytic cells. This procedure caused fibroblasts to predominantly attach to the dishes while most of the cardiomyocytes remained unattached. The population of cells enriched in cardiomyocytes was collected and counted. The cells were cultured in Dulbecco/Vogt modified Eagle's minimal essential medium (DMEM) with 1 mmol/L pyruvate, 10% foetal bovine serum (FBS), 100 units/mL penicillin, and 100 units/mL streptomycin. Using this protocol, > 95% of the cells were deemed cardiomyocytes as judged by sarcomeric myosin content. On the second day after plating, medium was replaced. Three to 4 days after plating, the cells were exposed to hyperglycaemic conditions. The high glucose-treated cardiomyocytes were generated by treating the cells with 30 mmol/L glucose for 24 h [15]. This animal experiment was also approved and conducted in accordance with local institutional guidelines for the care and use of laboratory animals in the Chi-Mei Medical Center (No. 100052307) and followed the Guide for the Care and Use of Laboratory Animals published by the US National Institutes of Health (NIH Publication No. 85-23, revised 1996), as well as the guidelines of the Animal Welfare Act.

### Blood pressure measurement

The systolic blood pressure (SBP) was monitored in rats with diabetes 8 weeks after induction. The blood pressure measurements in age-matched normal rats were designated as controls. The blood pressure of the tail artery was measured non-invasively with a photoelectric volume oscillometer (UR-5000, Ueda, Japan) by placing the tail cuff device around the tail of the rat. The measurements for SBP were recorded in quadruplicate for each rat and the average blood pressure was calculated.

### The effect of diazoxide (DZ) on heart rate on diabetic rats 8 weeks after induction

DZ (Sigma-Aldrich, Inc.), the well-known opener of K_ATP _channels[16], was iv injected at 0.5, 1, 2 mg/kg for diabetic rats and 3, 5, 8 mg/kg for normal rats. The changes in heart rates were recorded at 5 minutes interval for 30 minutes.

### The effects of insulin and phlorizin on diabetic rats 8 weeks after induction

Normalization of hyperglycemia was achieved per a previous protocol [17] in rats with diabetes for 8 weeks, using treatment either with 1 mg/kg of phlorizin dehydrate (Fluka Chemie, Buchs, Switzerland) or 0.5 IU of insulin (Novo Nordisk, Bagsvaerd, Denmark) by ip injection every 8 h for 4 days. Fluctuations in blood glucose levels were not observed in rats during repeated injections of insulin or phlorizin. Upon completion of treatment, the rats were sacrificed and their hearts were immediately removed, frozen in liquid nitrogen, and stored at -70°C until analysis was performed. The SUR-2A and Kir6.2 protein and mRNA concentrations in the heart tissue were measured by Western immunoblotting and Northern blotting, respectively. Blood samples were also collected from the femoral vein of the rats prior to sacrifice to estimate alterations in plasma glucose.

### Preparation of heart membrane fraction

The preparation of membrane fraction from whole heart was performed on ice. The isolated heart tissue was lysed in 10 ml of pH 7.4 Tris/EDTA buffer at 4°C and homogenized for 15 s. The membrane fraction was obtained by centrifugation at 20,000 g for 15 min.

### Measurement of K_ATP _channel protein

After homogenization, the protein content was determined by BioRad protein dye binding assay (Bio-Rad Laboratories, Richmond, CA, USA). Protein samples (9 μg) were separated by sodium dodecyl sulfate-polyacrylamide gel electrophoresis (10% acrylamide gel) using Bio-Rad Mini-Protein II system (Laemmli 1970). The separated proteins were blotted onto nitrocellulose. After treating with SUR-2A and Kir6.2 antibody (Affinity Bioreagents, Inc., Colorado, USA). Immunostaining was performed for peroxidase activity by incubation in Tris-buffer (10 mmol/l). The intensity of the blot incubated with goat polyclonal antibody (1:1000) to bind actin (Santa Cruz Biotechnology, Inc., Santa Cruz, CA, USA) was used to ensure that the amount of protein loaded into each lane of the gel was constant. Autoradiography was developed using an enhanced chemiluminescence development system (Amersham International, Buckinghamshire, UK). The resulting immunoblots were quantified by a scanning densitometer (Hoefer, San Francisco, CA, USA).

### Measurement of K_ATP _channel mRNA

Total RNA was extracted from heart using Ultraspec™-II RNA extraction system (Bioteck, Houston, TX, USA) as indication of the manufacturer. RNA (20 μg) was denatured and aliquots of total RNA were then size-fractionated in a 1.2% agarose/formaldehyde gel. The RNA was transferred to a Hybond-N membrane (Amersham International). SUR-2A and Kir6.2 mRNA levels were detected using prime-labeled full-length cDNA under stringent hybridization conditions [18]. Intensity of the mRNA blot was quantified by scanning densitometry (Hoefer, San Francisco, CA, USA). The response of β-actin was used as internal standard.

### Statistical analysis

Statistical analysis was carried out using ANOVA analysis and Newman-Keuls post-hoc analysis. Statistical significance was set as *p *< 0.05. Results were expressed as mean ± SE of each group from various samples (*n*).

## Results

### Changes of plasma glucose and systolic blood pressure in rats with diabetes for 8 weeks

The fasting plasma glucose levels of STZ-diabetic rats were significantly higher than those of untreated rats (Table 1). Additionally, the plasma insulin mean level in diabetic rats induced by STZ for 8 weeks was 13.4 ± 2.7 μU/ml (*n *= 8), markedly lower than the insulin level in control rats (58.3 ± 6.4 μU/ml; *n *= 8). Moreover, the plasma glucose levels in rats with diabetes for 8 weeks were markedly reversed by treatment with either insulin or phlorizin for 4 days, as compared to untreated diabetic rats (Table 1).

**Table 1:** Changes of body weight, plasma glucose level and hemodynamic parameters in 8 weeks STZ-diabetic rats received insulin (0

	Body weight (g/rat)	Plasma glucose (mg/dl)	Blood pressure (mmHg)	Heart rate (bpm)
Normal rats				
Vehicle-treated	252.6 ± 8.7	93.4 ± 7.5	119.4 ± 3.3	358.4 ± 9.3
Diabetic rats				
Vehicle-treated	182.3 ± 7.5**	348.2 ± 11.3***	96.3 ± 2.7*	320.2 ± 7.6*
Insulin-treated	189.4 ± 6.3**	223.6 ± 9.8**	114.2 ± 3.4	338.7 ± 10.2
Phlorizin-treated	184.6 ± 7.2**	248.1 ± 8.7**	118.4 ± 2.9	341.6 ± 8.3

Values are the mean ± SE for each group of 8 animals. * *P *< 0.05, ** *P *< 0.01, and *** *P *< 0.001 levels of significance, as compared to the values in normal rats that received treatment with vehicle in the same volume.

The SBP in diabetic rats induced by STZ for 8 weeks was significantly lower than that in age-matched normal rats. Injection of diabetic rats with 0.5 IU/kg insulin, three times daily for 4 days, restored the SBP to a level approaching normal control (Table 1). Similarly, treatment with phlorizin for 4 days elevated the SBP in rats with diabetes for 8 weeks to 118.4 ± 2.9 mmHg, which was not statistically different from the control rats (Table 1).

The body weight of STZ-diabetic rats was markedly less than that of normal control (Table 1). There was no difference in the body weight of diabetic rats between the vehicle- and insulin-treated groups throughout the 4-day study (Table 1). Also, phlorizin did not influence the body weight in STZ-diabetic rats throughout the 4-day treatment (Table 1).

### Effect of diazoxide (DZ) on the heart rate of rats

**Figure 1:**
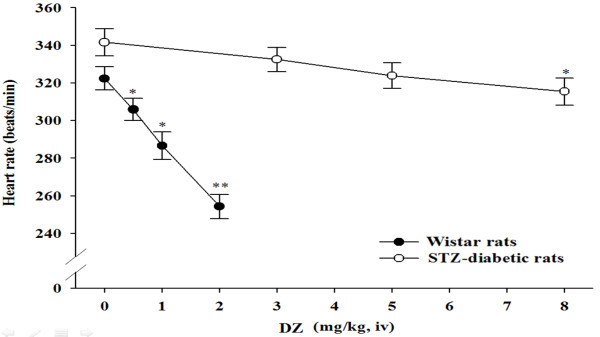
**Effects of K_ATP _channel opener, diazoxide (DZ), on the heart rates in STZ-diabetic rats with 8 weeks of diabetes (closed circles) and Wistar rats (open circles)**. Diabetic rats were iv injected with DZ at 0.5, 1 or 2 mg/kg. Wistar rats were received same treatment with DZ at 3, 5 or 8 mg/kg. The results are expressed as the mean ± SE obtained from each group of 7 animals. * *P *< 0.05 and ** *P *< 0.01 for comparisons of the post-injection heart rates with the pre-injection baseline heart rate (0) in each group.

Figure 1 showed that DZ reduced the heart rates in both STZ-diabetic and control rats. Also, heart rate decreased in STZ-diabetic rats (11.8 ± 2.4%) by DZ at the low dose of 0.5 mg/kg was more pronounced than that (2.6 ± 1.2%) in normal rats responding to 3 mg/kg DZ.

### Changes in mRNA levels of cardiac K_ATP _channels in rats with diabetes for 8 weeks

**Figure 2:**
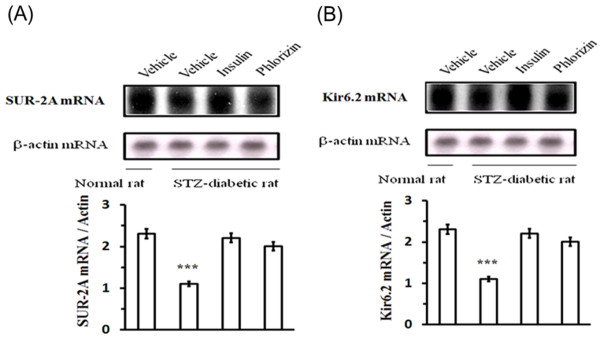
**Effects of insulin or phlorizin on the gene expression of cardiac K_ATP _channels in STZ-treated rats with 8 weeks of diabetes**. The upper panel is the autoradiograph resulting from Northern blotting of the cardiac SUR-2A (A), Kir6.2 (B) mRNA or β-actin in hearts isolated from these diabetic rats treated with insulin (0.5 IU) or phlorizin (1 mg/kg) three times daily for 4 days. The vehicle used to dissolve the test medications was given in the same volume. Similar results were obtained with an additional 5 replications. Quantification of mRNA levels using SUR-2A or Kir6.2/β-actin, expressed as mean with SE (n = 6 per group) in each column, is indicated in the lower panel. * *P *< 0.05, ** *P *< 0.01 and *** *P *< 0.001 vs. control.

The mRNA levels of cardiac SUR-2A and Kir6.2 were significantly decreased in diabetic rats that received STZ for 8 weeks as compared to the age-matched normal rats (Figure 2). This decrease in cardiac SUR-2A and Kir6.2 mRNA levels of rats with diabetes for 8 weeks was reversed after 4 days of insulin treatment to approximately the same level which existed in control rats. Treatment of these diabetic rats with 4 days of phlorizin also reversed the mRNA levels of SUR-2A and Kir6.2 in cardiac tissue to approximately 67% of the level compared with vehicle-treated diabetic rats (Figure 2).

### Changes in protein and mRNA levels of cardiac K_ATP _channels in rats with diabetes for 8 weeks

**Figure 3:**
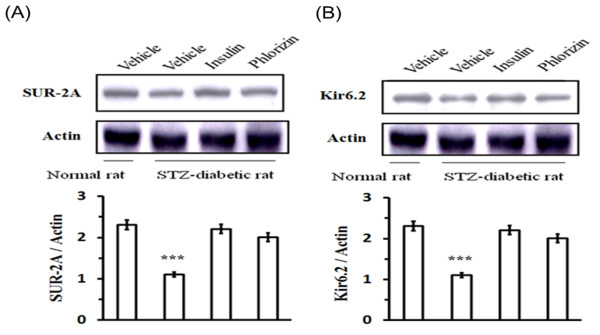
**Effects of insulin or phlorizin on the protein levels of cardiac K_ATP _channels in STZ-treated rats with 8 weeks of diabetes**. Upper panel depicts the Western blot analysis of representative protein levels for SUR-2A or Kir6.2, actin in hearts isolated from these diabetic rats treated in same manner. Quantification of protein levels using SUR-2A or Kir6.2/actin, expressed as mean with SE (n = 6 per group) in each column, is indicated in the lower panel. * *P *< 0.05, ** *P *< 0.01 and *** *P *< 0.001 vs. control.

The protein levels of cardiac SUR-2A and Kir6.2 in rats 8 weeks after STZ injection were markedly decreased to nearly 2-fold the levels in aged-matched controls (Figure 3). After 4 days of insulin injections, the cardiac SUR-2A and Kir6.2 protein levels in these diabetic rats were approximately 60% reversed than that in vehicle-treated diabetic rats (Figure 3). A marked recovery in the protein level of SUR-2A and Kir6.2 was also observed in the cardiac tissues of rats with diabetes for 8 weeks after a 4-day treatment of phlorizin, which was 64% of the level measured in the vehicle-treated diabetic rats (Figure 3).

### Decrease of K_ATP _channels expression levels by glucose in neonatal rat cardiac myocytes

**Figure 4:**
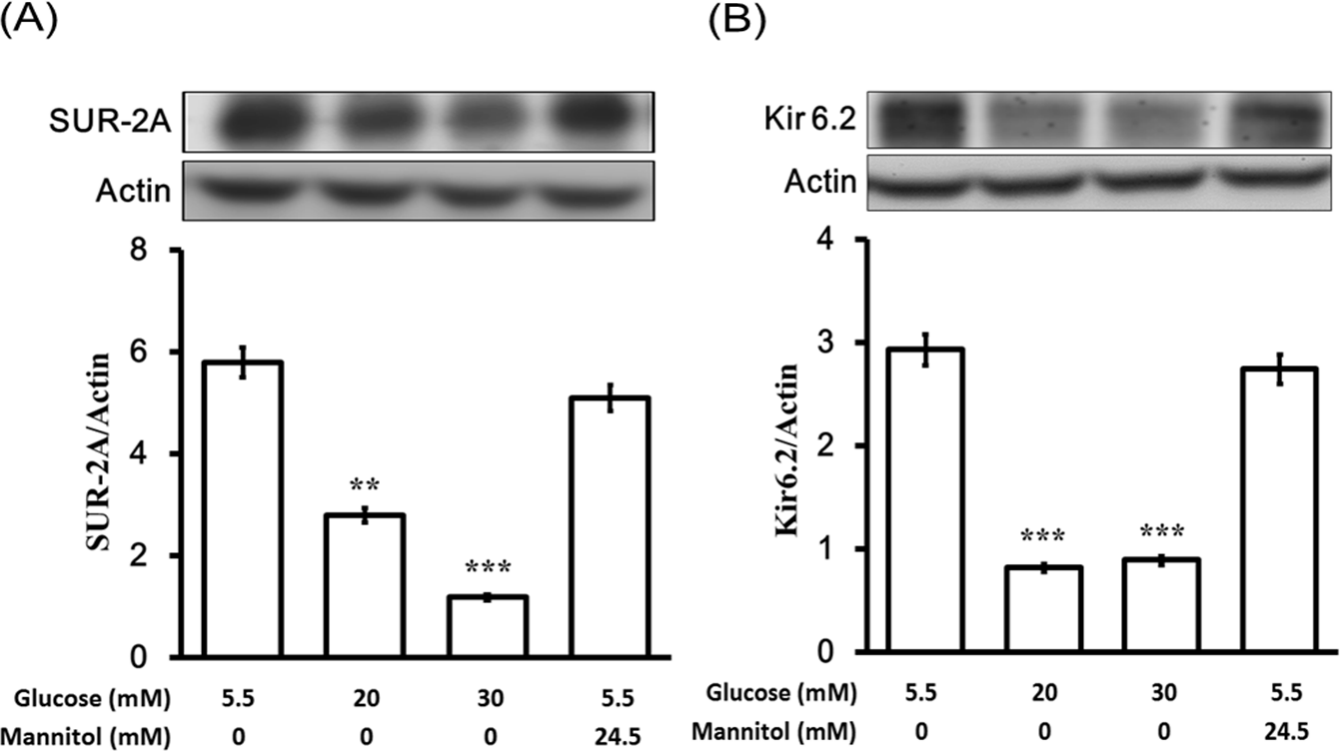
**Changes in expression of K_ATP _channels in neonatal rat cardiac myocytes after exposure to high concentrations of glucose**. Cultured neonatal rat cardiac myocytes exposed to glucose at final concentrations of 10 mM, 20 mM and 30 mM were used to compare with the control, which was incubated with 5.5 mM glucose. Cells were also exposed to 24.5 mM mannitol to produce the same osmolarity (317 mOsmol/L) as that produced when using the highest concentration of glucose (30 mM). After 24 hours of incubation, cells were prepared for Western blot analysis. Quantification of protein levels using SUR-2A or Kir6.2 over β-actin indicated as means with SE (n = 4 per group) in each column are presented in the lower panel. *P < 0.05, **P < 0.001 compared to control.

Effects of hyperglycemia on SUR-2A and Kir6.2 expression levels were further characterized by exposing cultured neonatal rat cardiac myocytes to various concentrations of glucose in vitro. The SUR-2A and Kir6.2 expression levels in neonatal rat cardiac myocytes were reduced in a concentration-dependent manner when cultured in media containing glucose after 24 hr incubation. The lack of change in SUR-2A and Kir6.2 expression levels in mannitol treated neonatal rat cardiac myocytes showed that protein expression levels were not affected by hyperosmolarity. Thus, we identified that expression levels of SUR-2A and Kir6.2 are decreased by high glucose in neonatal rat cardiac myocytes, similar to the changes observed in heart of diabetic rats (Figure 4).

### Restored of *K_ATP _channels *expression levels in high-glucose treated neonatal rat cardiac myocytes by free radicals scavenger

**Figure 5:**
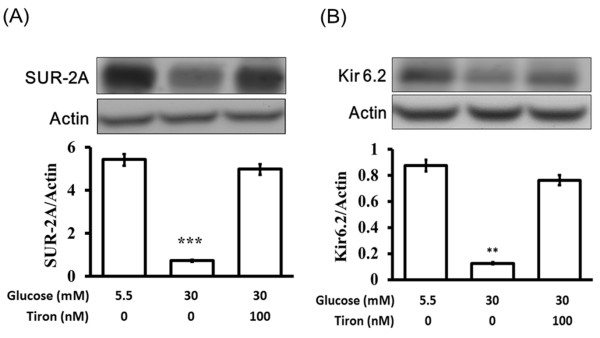
**Effects of tiron on the protein levels of cardiac K_ATP _channels in high-glucose treated neonatal rat cardiac myocytes**. Cultured neonatal rat cardiac myocytes exposed to glucose at final concentrations of 30 mM were used to compare with the control, which was incubated with 5.5 mM menitol. Cells were also exposed to 100 nM tiron as radical scavenger. After 24 hours of incubation, cells were prepared for Western blot analysis. Quantification of protein levels using SUR-2A or Kir6.2 over β-actin indicated as means with SE (n = 4 per group) in each column are presented in the lower panel. * *P *< 0.05, ** *P *< 0.001 compared to control.

Free radicals (ROS) are excessively produced in the pathogenesis of acute and diseases[19]. Present studies have demonstrated that cardiac dysfunction is caused by increasing of ROS production in hyperclycemic rats and high-glucose treated cardiac myocytes. To investigate the effect of hyperglycemia on SUR-2A and Kir6.2 expression, we treated neonatal rat cardiac myocytes with high concentrations of glucose (30 mmol/l; HG) in medium with and without 100 nmol of tiron, a superoxide anion scavenger[20] and after incubation we examined the effects on SUR-2A and Kir6.2 expression using western blot analysis (Figure 5). We observed that the decreased SUR-2A and Kir6.2 protein expressions in neonatal rat cardiac myocytes caused by high glucose concentrations were reversed by tiron treatment (Figure 5); these are consistent with our previous observations of SUR-2A and Kir6.2.

## Discussion

Due to the difference between human and animal and/or the stage of diabetes, the response of blood pressure is varied; changes of blood pressure in diabetic complications seem not so simple. Numerous evidences suggest that diabetic heart is characterized by compromised ventricular contraction and prolonged relaxation attributable to multiple causative factors including calcium accumulation, oxidative stress and apoptosis. Previous study have demonstrated that blocking the calcium channel and oxidative stress could have advantage in diabetic heart [21]. Another study also showed that hyperglycemia can cause systolic dysfunction and a higher expression of cTnI in cardiomyocytes through ROS, enhancing MEK/ERK-induced GATA-4 phosphorylation and accumulation in the cell nucleus [22]. Although chronic diabetes is commonly linked to hypertension[23], hypotension has been ubiquitously described in early stage of diabetes[24,25]. In the present study, cardiac dysfunction as evidenced by bradycardia and hypotension has been observed early in the course of diabetic rats receiving STZ for 8 weeks. Similar cardiac pathology in spontaneously diabetic Bio-Breeding rats has also been reported; specifically, the heart rate and heart rate variability were significantly lower than the control rats [26].

We showed that the mRNA level of cardiac K_ATP _channels in rats with diabetes for 8 weeks duration was markedly lower than in non-diabetic rats. Also, changes in the protein level of cardiac K_ATP _channels were associated with steady-state levels of mRNA encoding this receptor. A decrease of cardiac K_ATP _gene expression was observed during the early stage of type-1 like diabetes. It is well known that the most prominent role of K_ATP _channels in cardiovascular system is that opening of this channel can protect cardiac myocytes against ischemic injuries [27]. Actually, the effect of diazoxide (DZ) through opening of K_ATP _channels was also decreased in rats with diabetes for 8 weeks duration. Therefore, decreased expression of cardiac K_ATP _channel is one of the mechanisms accounting for cardiac dysfunction in the early stage of diabetes.

Several mechanisms have been proposed to explain the pathogenesis of diabetic complications, and hyperglycemia is always implicated [28]. Abnormal sympathetic nervous system and β-adrenoceptor (β-AR) signaling is associated with diabetes. β-AR have been found reduced the expression under Hyperglycemia [29]. In an attempt to know the role of hyperglycemia and/or hypoinsulinemia in the changes of cardiac K_ATP _channels in insulin-deficient diabetic rats, exogenous insulin was administrated for 4 days into the diabetic rats, 8 weeks following induction with STZ. We found that insulin treatment of diabetic rats reversed the blood pressure reduction. In addition, normalization of plasma glucose level with insulin had a tendency to reverse the lower expression of cardiac K_ATP _channels in 8 weeks diabetic rats. Phlorizin is an inhibitor of the renal tubular reabsorption of glucose and it has been widely used to distinguish the role of hyperglycemia in STZ-diabetic rats [30]. The reductions in blood pressure as well as the lowerer gene expression of cardiac K_ATP _in these diabetic rats were also reversed by the reduction of hyperglycemia from phlorizin injection. Therefore, hyperglycemia is related to the down-regulation of cardiac K_ATP _channels during the early stage of diabetes. In the present study, similar changes of K_ATP _channels were observed on the whole heart of experimental diabetes, instead of limiting to atria or ventricles. Hyperglycemia could be considered a key in cardiac alteration that was associated with the decrease in cardiac K_ATP _channels gene expression, leading to result in hypotension observed in 8 weeks type-1 diabetic rats.

We also demonstrated that expression levels of SUR-2A and Kir6.2 are decreased by high glucose in neonatal rat cardiac myocytes, similar to the changes observed in heart of diabetic rats. Furthermore, in cultured neonatal rat cardiac myocytes, the reduced expressions of SUR-2A and Kir6.2 caused by high concentrations of glucose were also reversed by the antioxidant tiron.

Clinically, heart disease is one of the major causes of death in diabetic patients, due in part to the accumulation of advanced glycation end products (AGE) resulting from chronic hyperglycemia [31]. AGE is known to produce from various pathways such as lipid peroxidation or oxidative stress, in addition to the underlying glycemia and accumulation in blood and tissues at an extremely accelerated rate, which is correlated with the time course of diabetes [32]. It has been suggested that suitable glycemic control in patients with diabetes for 8 years does not lead to an effective reduction in AGE levels, illustrating the negative correlation between hyperglycemia and the advanced diabetic complications that occur in chronic diabetes[32]. It appears that hyperglycemia may decrease cardiac K_ATP _channels gene expression to account for the changes of cardiovascular function during the early stage of diabetes [28]. Thus, more studies are necessary to clarify the detailed mechanism(s) in the near future. The current study supports the recommendations for glycemic control of diabetes at early stage to lower complications.

## Conclusions

We have demonstrated that both mRNA and protein expression of cardiac K_ATP _channels are decreased in diabetic rats induced by STZ for 8 weeks. This phenomenon leads to reduction of blood pressure. Correction of hyperglycemia by insulin or phlorizin restored the gene expression of cardiac K_ATP _in these diabetic rats. These data suggest that an increase of plasma glucose may enhance cardiac K_ATP _channels gene expression to result in cardiac dysfunction observed in diabetic rats.

## List of abbreviations

KATP: ATP-sensitive potassium channels; HR: heart rate; STZ: streptozotoxin; CO: cardiac output; i.p.: intraperitoneal; HG: high glucose; PBS: phosphate-buffered saline; BSA: bovine serum albumin; RIPA: radioimmunoprecipitation assay; SDS-PAGE: sodium dodecyl sulfate polyacry lamide gel electrophoresis; SBP: systolic blood pressure; SD: standard deviation; SEM: standard error of mean; DZ: Diazoxide; SUR2A: sulfonylurea receptors2A; kir 6.2: inward-rectifier potassium ion channels 6.2; IV: intravenous; FBS: foetal bovine serum; ROS: reactive oxygen species.

## Competing interests

The authors declare that they have no competing interests.

## Authors' contributions

JTC, LJC and KCC carried out the molecular studies and drafted the manuscript. YZC and YXL were involved in the interpretation of the results. ZCC and JTC conceived the study and participated in its design, interpretation and coordination, and drafted and approved the manuscript. All authors have read and approved the final manuscript. Also, all authors contributed significantly to and are in agreement with the content of the manuscript.
